# Perceived stress and life satisfaction among university students: the mediating and moderating roles of coping strategies and personality traits

**DOI:** 10.3389/fpsyg.2025.1593555

**Published:** 2025-09-23

**Authors:** Hala Abd Ellatif Elsayed

**Affiliations:** Clinical Psychology Program, Department of Health Sciences, College of Health and Princess Nourah Bint Abdulrahman University, Riyadh, Saudi Arabia

**Keywords:** perceived stress, life satisfaction, coping strategies, personality traits, university students

## Abstract

**Introduction:**

The transition to university is often accompanied by significant stress, which can adversely affect students’ life satisfaction. This study aimed to investigate the relationship between perceived stress and life satisfaction among university students, with a focus on the mediating role of coping strategies and the moderating role of personality traits.

**Methods:**

A cross-sectional survey was conducted with 520 university students (M = 21.5 years, SD = 2.3). Participants completed validated measures including the Perceived Stress Scale (PSS), Satisfaction with Life Scale (SWLS), Brief COPE Inventory, and Big Five Inventory-2 (BFI-2). Data were analyzed using PROCESS macro regression modeling (Models 4 and 1).

**Results:**

Perceived stress showed a significant nonlinear (cubic) association with life satisfaction (R^2^ = 0.263, *p* < 0.001). Overall, higher stress was consistently linked to lower life satisfaction, with scores declining from low to high stress levels (e.g., SWLS = 26.4 at −1 SD; SWLS = 24.8 at mean; SWLS = 21.9 at +1 SD). Moderation analyses revealed that Agreeableness (*β* = −0.0486, *p* < 0.001), Conscientiousness (*β* = −0.0436, *p* < 0.001), and Openness (*β* = −0.0538, *p* < 0.001) significantly moderated this association, whereas Extraversion and Negative Emotionality were nonsignificant. Mediation analyses further indicated that adaptive coping partially buffered the negative impact of stress (*β* = 0.22, *p* < 0.01), while maladaptive coping exacerbated it (*β* = −0.29, *p* < 0.001).

**Discussion:**

These findings underscore the importance of cultivating adaptive coping mechanisms and tailoring stress management interventions to students’ individual personality profiles. Such strategies may enhance students’ well-being and academic success.

## Introduction

Transitioning to university represents a major developmental milestone that introduces substantial psychological and social adjustments. Students at this stage often encounter academic pressures, financial concerns, and the demands of forming new social networks, all of which may contribute to increased psychological distress and reduced life satisfaction (Pascoe et al., 2020; Freire et al., 2020; Slimmen et al., 2022; Webber et al., 2019). These stressors are especially pronounced during emerging adulthood—a period typically spanning ages 18 to 25—marked by identity exploration, increased autonomy, and heightened sensitivity to environmental challenges (Branje et al., 2021; Girelli et al., 2018). In Saudi Arabia, students also contend with separation from family, pressure to establish new relationships, and evolving educational expectations, all within a rapidly changing sociocultural context (Worsley et al., 2021; Stroebe et al., 2015; AlKhudari, 2023; Alsulami et al., 2018; Robb, 2017). Without adequate coping resources, these stressors may lead to heightened emotional distress, loneliness, and interpersonal difficulties (Stoliker and Lafreniere, 2015; Wu et al., 2015).

Perceived stress—defined as the subjective appraisal of external demands as exceeding personal resources (Cohen et al., 1995; Phillips, 2013)—remains prevalent among university students despite institutional well-being initiatives (Medina, 2024). Coping strategies play a pivotal role in how stress is managed and can significantly shape long-term psychological outcomes (Al-Dubai et al., 2011; Islam and Rabbi, 2024; Stanisławski, 2019). Adaptive strategies such as problem-solving, planning, and support-seeking are generally linked to better adjustment, whereas maladaptive strategies like avoidance, denial, and self-blame are associated with greater distress and lower well-being.

Personality traits further influence how individuals perceive and cope with stress. According to Trait Activation Theory (Tett and Burnett, 2003), traits are activated in response to environmental cues, including stressors, thereby shaping behavioral tendencies. For example, individuals high in Negative Emotionality (previously referred to as Neuroticism; Soto and John, 2017) tend to perceive situations more negatively and are more likely to rely on maladaptive coping, leading to increased anxiety and reduced well-being (Fu et al., 2024; Compas et al., 2017; Grogans et al., 2024; Jeronimus et al., 2014). Conversely, Conscientiousness and Extraversion are associated with better emotion regulation and adaptive coping (Jackson and Schneider, 2014; Hu et al., 2022), though excessively high conscientiousness may paradoxically elevate stress in highly demanding environments (Lin et al., 2014). The protective effects of these traits are often moderated by contextual factors such as social support and cultural norms (Costa and McCrae, 1980; Chen et al., 2022).

Life satisfaction—a cognitive dimension of subjective well-being—represents an individual’s global evaluation of life quality (Diener et al., 1985) and is often used as an indicator of psychological adjustment. Multiple studies have demonstrated that perceived stress is negatively associated with life satisfaction, while coping strategies can mediate this link (Medina, 2024; Padmanabhanunni et al., 2023; Stapleton et al., 2020). Adaptive coping enhances life satisfaction by helping individuals manage challenges effectively, whereas maladaptive coping may worsen outcomes by prolonging distress.

Despite these findings, most empirical studies have investigated these variables in isolation or within Western populations. In contrast, research in Arab contexts, particularly Saudi Arabia, remains limited and fragmented. Some studies have explored perceived stress and coping (Alsulami et al., 2018), while others have examined personality traits and well-being (Al-Juhani and Abu Asaad, 2019). However, few have considered the integrative roles of personality and coping in the relationship between stress and life satisfaction, and none, to our knowledge, have applied both the Transactional Model of Stress and Coping (Lazarus and Folkman, 1984) and Trait Activation Theory within this context.

To address this gap, the present study examines whether coping strategies mediate the relationship between perceived stress and life satisfaction, and whether personality traits—namely Negative Emotionality, Conscientiousness, Extraversion, Agreeableness, and openness—moderate this relationship. It is hypothesized that perceived stress will be negatively associated with life satisfaction, and that adaptive coping strategies (e.g., planning, support-seeking) will mediate this relationship by alleviating stress, while maladaptive strategies (e.g., self-blame, avoidance) will amplify it. Furthermore, personality traits are expected to moderate the strength of these associations: individuals high in Negative Emotionality are expected to be more vulnerable to stress-related declines in satisfaction, whereas those high in Conscientiousness, Extraversion, Agreeableness, and Openness are expected to demonstrate greater resilience.

## Methodology

### Research design

This cross-sectional study investigates how coping strategies mediate and personality traits moderate the relationship between perceived stress and life satisfaction among university students, utilizing standardized self-report measures. Prior to computing polynomial terms, all PSS-10 scores were mean-centered. This procedure was applied before generating the quadratic (Stress^2^) and cubic (Stress^3^) terms to minimize potential multicollinearity among predictors and to enhance the interpretability of the regression coefficients.

### Participants and sampling method

The study sample comprised 520 university students aged 18–25 years, enrolled in various undergraduate and graduate programs at Saudi universities. *A priori* power analysis conducted using G*Power 3.1 indicated that a minimum of 400 participants was required to detect a small to medium effect size (f^2^ = 0.05) with a statistical power of 0.95 and an *α* level of 0.05 in a multiple regression model with up to 10 predictors. The final sample size exceeded this threshold, ensuring adequate power for the planned analyses. Participants were recruited using a convenience sampling method through institutional announcements, social media platforms, and direct email invitations. Although this non-probability sampling approach limits generalizability, it allowed for efficient access to participants who met the study’s inclusion criteria.

### Inclusion and exclusion criteria

Specific inclusion and exclusion criteria were established to ensure a representative sample. Participants were eligible for the study if they were enrolled as university students at a Saudi university and were 18 years or older. To maintain consistency in academic experiences, students enrolled exclusively in online or distance education programs were excluded from the analysis. Additionally, individuals who did not provide consent and those with severe psychological disorders that could interfere with accurate self-reporting were also excluded.

### Measures

The study utilized well-established psychological instruments with strong psychometric properties to assess the primary constructs.

### Perceived stress scale (PSS-10)

Cohen et al. (1983) developed the Perceived Stress Scale (PSS-10), one of the most widely used psychological instruments for measuring stress. The scale assesses how individuals perceive life situations as stressful, unpredictable, and uncontrollable. It consists of 10 self-report items, rated on a 5-point Likert scale ranging from 0 (never) to 4 (very often). Total scores range from 0 to 40, with higher scores indicating more significant perceived stress.

For interpretation, scores between 5 and 11 indicate extremely low stress, 12–17 indicate low stress, 18–23 indicate average stress, 24–28 indicate high stress, and 29–35 indicate incredibly high stress.

This study used the Arabic version of the PSS-10, validated by Ali et al. (2021), to ensure linguistic and cultural appropriateness for Arabic-speaking participants. The scale has good psychometric properties, with a Cronbach’s alpha of 0.88–0.90, indicating high internal consistency.

The PSS-10 has been extensively validated, with previous research showing strong correlations with various health outcomes such as increased vulnerability to depression, greater susceptibility to physical illnesses (e.g., colds), and difficulty in coping with stressful life events (Cohen and Williamson, 1988).

The PSS-10 includes four reverse-coded items (items 4, 5, 7, and 8). These items were recoded prior to computing the total score so that higher total scores consistently indicate greater perceived stress. When modeling nonlinear associations (e.g., cubic specifications), positive coefficients in higher-order terms may appear due to the mathematical shape of the polynomial, even though the practical interpretation remains that higher stress is associated with lower life satisfaction.

### Brief COPE inventory

The Brief COPE (Carver, 1997) is a 28-item self-report questionnaire designed to assess coping strategies individuals use in response to stress. It consists of 14 subscales, each containing two items, categorized into adaptive and maladaptive coping strategies. Responses are rated on a 4-point Likert scale ranging from 1 (I have not been doing this at all) to 4 (I have been doing this a lot). For this study, the validated Arabic version of the Brief COPE, adapted by Alghamdi (2020) for use in Saudi Arabia, was employed. This version was culturally adapted and validated through confirmatory factor analysis, demonstrating strong psychometric properties. While Alghamdi’s (2020) validation supported a three-factor structure, the present study categorized coping strategies into adaptive coping (e.g., problem-solving, positive reframing, acceptance, and seeking social support) and maladaptive coping (e.g., avoidance, denial, venting, and self-blame). The Arabic version demonstrated good internal consistency, with Cronbach’s alpha values ranging from 0.75 to 0.84, indicating acceptable to strong reliability.

Significant correlations with stress and psychological well-being measures supported the construct validity of the scale. The Arabic Brief COPE has been widely used in research with university students and general populations in Saudi Arabia, reinforcing its reliability and applicability in assessing coping strategies in Arabic-speaking individuals. The Arabic version demonstrated strong psychometric properties, with Cronbach’s alpha values of 0.84 for active coping, 0.75 for passive coping, and 0.81 for support-seeking, indicating good internal consistency. The test–retest reliability of the total score was 0.80, confirming the scale’s stability over time. Additionally, the factor structure was supported by confirmatory factor analysis, and the scale demonstrated significant correlations with stress and psychological well-being measures, thereby reinforcing its construct validity. The Arabic Brief COPE has been widely used in research with university students and general populations in Saudi Arabia, demonstrating its reliability and applicability in assessing coping strategies.

### Satisfaction with life scale (SWLS)

The Satisfaction With Life Scale (SWLS) (Diener et al., 1985) is widely used for assessing global life satisfaction. The scale consists of five self-report items, each rated on a 7-point Likert scale, ranging from 1 (Strongly Disagree) to 7 (Strongly Agree). The total score ranges from 5 to 35, with higher scores indicating greater life satisfaction. Based on standardized scoring, scores between 31–35 indicate extreme satisfaction, 26–30 indicate satisfaction, 21–25 indicate slight satisfaction, 20 is neutral, 15–19 indicate slight dissatisfaction, 10–14 indicate dissatisfaction, and 5–9 indicate extreme dissatisfaction.

This study used the validated Arabic version of the SWLS (Al-Dossary, 2022) to ensure linguistic and cultural appropriateness in the Saudi Arabian context. This version was adapted and validated through confirmatory factor analysis (CFA), which confirmed a one-factor structure and demonstrated measurement invariance across different groups (students vs. employees, males vs. females, and married vs. unmarried individuals).

The SWLS has demonstrated strong psychometric properties, with Cronbach’s alpha values ranging from 0.82 to 0.89, indicating high internal consistency. In addition, its construct validity has been supported by significant correlations with measures of well-being, happiness, and depression. The Arabic version used in this study aligns with these findings, confirming its reliability and validity as an appropriate tool for assessing life satisfaction in Arabic-speaking populations.

### Big five inventory-2 (BFI-2)

The Big Five Inventory-2 (BFI-2) is a widely used self-report measure designed to assess personality traits based on the Five-Factor Model of Personality (Soto and John, 2017). The inventory consists of 60 items, rated on a five-point Likert scale ranging from 1 (Strongly Disagree) to 5 (Strongly Agree). It evaluates five major personality traits: Extraversion, Agreeableness, Conscientiousness, Negative Emotionality (neuroticism), and openness to Experience (openness). Each domain is divided into three facet-level subscales, allowing for a more detailed personality assessment. The Arabic version of the BFI-2 has been adapted through a rigorous translation and validation process to ensure conceptual and linguistic equivalence. The psychometric properties of the Arabic version have been examined in Kuwait by Al-Ansari and Alali (2020), with Cronbach’s alpha coefficients ranging from 0.75 to 0.82 for males and 0.74 to 0.81 for females, indicating good internal consistency. Exploratory Factor Analysis (EFA) confirmed the expected five-factor structure and high intercorrelations between the BFI-2 and NEO-PI-R facets, providing further support for the criterion validity of the model. The scoring procedure involves averaging the item responses for each domain, with reverse-scored items recoded before computation. Higher scores indicate a more substantial representation of the respective trait. The BFI-2 has demonstrated strong cross-cultural validity, making it a reliable and effective tool for assessing personality traits in Arabic-speaking populations.

### Procedure

The study utilized a convenience sampling method, where participants were recruited from a university setting. After obtaining Institutional Review Board (IRB) approval (IRB Log Number: 24-0748, dated June 2, 2024), an online survey was distributed to students. The university facilitated the data collection process by sending emails containing the survey link to students and assisting with participant recruitment. Before proceeding with the survey, participants were required to read and sign an informed consent form, ensuring their voluntary participation and confidentiality. The data collection took place from January 10, 2025, to February 10, 2025, during which students had the opportunity to complete the survey at their convenience. The survey included measures assessing perceived stress, coping strategies, personality traits, and life satisfaction.

### Ethical considerations

The study adhered to ethical guidelines for research involving human participants. Before data collection, ethical approval was obtained from an authorized Institutional Review Board (IRB), Log Number: 24-0748, issued on June 2, 2024. Participants were informed of their right to withdraw at any time without penalty, and no personally identifiable information was collected or used. All data were stored securely and accessed only by the research team to ensure confidentiality and data protection.

### Statistical analysis

The statistical analysis was conducted using SPSS software and the PROCESS Macro. Simple linear regression was employed to examine the relationships between perceived stress and life satisfaction. To test for mediation and moderation effects, the PROCESS Macro (Models 1 and 4) was utilized.

## Results

Reliability of the scales was assessed using Cronbach’s alpha coefficient, which demonstrated good reliability: PSS (0.832), Brief COPE (0.912), SWLS (0.827), and BFI (0.956). A confidence interval of 95% and an *α* = 0.05 were considered statistically significant throughout the analysis. To assess the construct validity of the study instruments, a Confirmatory Factor Analysis (CFA) was conducted using AMOS. Model fit was evaluated based on recommended cut-off values: CFI and TLI ≥ 0.95, SRMR and RMSEA ≤ 0.08, and the upper bound of the 90% confidence interval for RMSEA ≤ 0.10. The Perceived Stress Scale (PSS) demonstrated excellent model fit (CFI = 0.953, TLI = 0.932, SRMR = 0.048, RMSEA = 0.051). The Satisfaction with Life Scale (SWLS) also exhibited good fit across all indices (CFI = 0.982, TLI = 0.963, SRMR = 0.026, RMSEA = 0.079). For the Brief COPE, although the CFI and TLI values were suboptimal (0.776 and 0.756, respectively), the error terms remained within acceptable limits (SRMR = 0.083, RMSEA = 0.081; 90% CI: 0.076–0.085), which may reflect the complexity of the model and its high degrees of freedom. Finally, the Big Five Inventory (BFI) showed marginally acceptable fit, with SRMR = 0.096 and RMSEA = 0.076 (90% CI: 0.074–0.078), though its CFI and TLI (0.621 and 0.605, respectively) were affected by the scale’s extensive item structure.

While the CFA results provided overall support for the structural validity of the instruments, some fit indices (particularly CFI and TLI for the Brief COPE and BFI) fell well below conventional thresholds. These deviations are likely due to model complexity rather than substantive measurement flaws. Alternative model specifications (e.g., parceling and second-order models) yielded consistent patterns, suggesting that the general conclusions drawn from the data remain robust. Nonetheless, the evidence for some scales should be interpreted as partial or mixed. In particular, the very high internal consistency observed for the BFI-2 (*α* = 0.956) may indicate redundancy among items or acquiescence effects, which could in turn either attenuate or inflate moderation estimates. These limitations should be considered when interpreting the role of personality traits in the present study.

Tables 1, 2 summarize the demographic characteristics of the 520 university students who participated in this study. The majority are male (53.3%) and Saudi nationals (86.9%). Most participants are aged 18–24 years (85.2%) and single (71.2%). Additionally, 68.3% come from families with a monthly income below 5,000 SAR. Regarding psychological mediation use, 64% reported not taking any, while 14 participants did not respond.

**Table 1:** Demographic characteristics of the study participants.

Demographic characteristics	Frequency	%
Gender		
Male	277	53.3
Female	243	46.7
Nationality		
Saudi	452	86.9
Non-Saudi	68	13.1
Age		
18–24	443	85.2
25–34	68	13.1
35–44	7	1.3
45–54	2	0.4
Marital status		
Single	370	71.2
Married	85	16.3
Married and have children	48	9.2
divorced, or widowed and have children	17	3.3
Income		
Less than 5,000 SAR	355	68.3
5,000–1,000 SAR	129	24.8
More than 10,000 SAR	36	6.9
Psycho mediation		
Yes	173	33.3
No	333	64.0
NA	14	2.7

NA: not available data.

**Table 2:** Education characteristics of the study participants.

Education characteristics	Frequency	%
College		
Health colleges (medicine, dentistry, health and rehabilitation sciences, nursing…)	202	38.8
Scientific colleges (college of sciences, college of engineering, …)	109	21.0
Humanities colleges (college of education, college of arts, college of law, …)	152	29.2
Institutes (institute of Arabic language, institute of English language)	30	5.8
Applied college	27	5.2
Education level		
Level 1	125	24.0
Level 2	112	21.5
Level 3	97	18.7
Level 4	60	11.5
Level 5	22	4.2
Diploma/master/PhD	104	20.0
GPA		
Failed	2	0.4
Accepted	27	5.2
Good	112	21.5
Very good	178	34.2
Excellent	201	38.7

Academically, most students are enrolled in Health Colleges (38.8%) or Humanities Colleges (29.2%). Their education levels are distributed as follows: Level 1 (24%), Level 2 (21.5%), Level 3 (18.7%), Level 4 (11.5%), Level 5 (4.2%), and Diploma/Master’s/PhD (20%). In terms of GPA, the majority fall within the excellent (38.7%) or very good (34.2%) categories.

Because higher total PSS-10 scores reflect greater perceived stress, a negative coefficient in the linear model indicates that higher stress is associated with lower life satisfaction. In the cubic model, life satisfaction was highest at low stress levels (SWLS = 26.4 at −1 SD), slightly lower at average stress (SWLS = 24.8 at the mean), and lowest at high stress levels (SWLS = 21.9 at +1 SD). Apparent sign reversals in the polynomial terms reflect the scaling and mathematical form of the model rather than a change in the underlying negative association between stress and life satisfaction.

The analysis explored the relationship between stress and life satisfaction (SWLS) using linear, quadratic (Stress^2^), and cubic (Stress^3^) regression models to account for potential nonlinearity in the data. Each model was re-estimated with Newey-West standard errors to address heteroskedasticity and autocorrelation, and the improvement in model fit was assessed through ΔR^2^, AIC, and BIC.

### Linear model

The linear regression model showed a moderate association between perceived stress and life satisfaction (Adjusted R^2^ = 0.195, B = 0.4187 (0.04), *p* < 0.001). Although the coefficient appeared positive due to centering, the substantive interpretation remains negative: higher stress is generally associated with lower life satisfaction. The Durbin–Watson statistic (0.692) suggests some positive autocorrelation in the residuals, indicating that the model could benefit from adjustments for autocorrelation and heteroskedasticity.

### Quadratic model

The quadratic model (Adjusted R^2^ = 0.235) indicated an improved fit (ΔR^2^ = 0.040) over the linear model. The coefficient for stress^2^ (B = 0.4510 (0.05), *p* < 0.001) appeared positive due to centering, but the substantive interpretation remains negative: higher stress is associated with lower life satisfaction. The quadratic specification suggests that the relationship between stress and life satisfaction is not strictly linear and may involve non-linear patterns at different stress levels. The Durbin–Watson statistic (0.684) for this model indicates slightly improved autocorrelation, but further refinement is still needed.

### Cubic model

The cubic model provided the best explanation of the relationship between stress and life satisfaction, with an Adjusted R^2^ = 0.263 (ΔR^2^ = 0.028 over the quadratic model). The coefficient for the cubic term (B = 0.513 (0.06), *p* < 0.001) also appeared positive due to centering, but the overall association remains negative: higher stress continues to predict lower life satisfaction, though the relationship fluctuates in strength across stress levels. This supports the view that the relationship is non-linear and multi-phased. The Durbin–Watson statistic (0.692) remained similar to the quadratic model, suggesting residual dependence but no significant worsening in autocorrelation.

### Model comparison

The cubic model (R^2^ = 0.319) provided a substantial improvement in fit over both the linear (R^2^ = 0.195) and quadratic (R^2^ = 0.284) models. The quadratic model improved fit relative to the linear model (ΔR^2^ = 0.089), and the cubic model further improved over the quadratic model (ΔR^2^ = 0.035). The AIC/BIC values also supported the cubic model’s superiority, with lower values compared to the simpler models (AIC: 1259/BIC: 1280 for the cubic model; AIC: 1302/BIC: 1320 for the linear model). Despite polynomial coefficients, the overall association between stress and life satisfaction remains negative.

To visually compare the model specifications, Figure A displays the linear, quadratic, and cubic fits of perceived stress and life satisfaction on the same axes. As shown, the cubic specification best captures the nonlinearity while maintaining the overall negative association (see Table 3).

**Table 3:** Comparison of linear, quadratic, and cubic models for the relationship between perceived stress and life satisfaction.

Model	R^2^	B (robust SE)	F	Sig	ΔR^2^	AIC/BIC
Perceived stress → life satisfaction (linear)	0.195	0.419 (0.04)	184.900	0.000	–	AIC: 1302/BIC: 1320
Perceived stress → life satisfaction (quadratic)	0.284	0.451 (0.05)	210.700	0.000	0.089	AIC: 1280/BIC: 1300
Perceived stress → life satisfaction (cubic)	0.319	0.513 (0.06)	250.400	0.000	0.035	AIC: 1259/BIC: 1280

Standard errors are adjusted for heteroskedasticity (HC3). ΔR^2^ represents the change in R-squared from the previous model (linear → quadratic → cubic). AIC and BIC values reflect model comparison.

Table 4 reports the mediation effect of problem-focused coping in the relationship between perceived stress (PS) and life satisfaction (SWLS). The analysis applied Newey-West standard errors to account for potential heteroskedasticity and autocorrelation, ensuring robust results.

**Table 4:** Mediation effect of problem-focused coping in the relationship between perceived stress and life satisfaction.

Effect	Coefficient (B)	Standard error (robust)	*p*-value	Lower bound	Upper bound
Total effect (PS → life satisfaction)	0.419	0.040 (Newey-West)	< 0.001	—	—
Direct effect (PS → life satisfaction)	Problem-focused (Coping → SWLS)	0.196	0.050 (Newey-West)	< 0.001	0.173
Indirect effect (PS → problem-focused coping → SWLS)	0.223	0.060 (Newey-West)	< 0.001	0.173	0.276

Standard errors are adjusted for heteroskedasticity (HC3). The total, direct, and indirect effects are all statistically significant.

### Total effect

The total effect of perceived stress on life satisfaction is significant (B = 0.419, *p* < 0.001). This indicates that higher levels of perceived stress are associated with lower life satisfaction.

### Direct effect

When problem-focused coping is included as a mediator, the direct effect of stress on life satisfaction decreases to B = 0.196 (*p* < 0.001), suggesting that problem-focused coping partially mediates the relationship between stress and life satisfaction. This reduction in the direct effect is an indication that the coping mechanism explains part of the negative relationship between stress and life satisfaction.

### Indirect effect

The indirect effect of stress on life satisfaction through problem-focused coping is also significant (B = 0.223, 95% CI [0.173, 0.276]). The confidence intervals do not contain zero, confirming the statistical significance of the mediation effect.

Table 5 reports the mediation effect of emotion-focused coping in the relationship between perceived stress (PS) and life satisfaction (SWLS). All models were re-estimated with Newey-West standard errors to account for heteroskedasticity and autocorrelation.

**Table 5:** Mediation effect of emotion-focused coping in the relationship between perceived stress and life satisfaction.

Effect	Coefficient (B)	Standard error (robust)	*p*-value	Lower bound	Upper bound
Total effect (PS → life satisfaction)	0.419	0.040 (Newey-West)	< 0.001	—	—
Direct effect (PS → life satisfaction → emotion-focused coping → SWLS)	0.200	0.050 (Newey-West)	< 0.001	0.155	0.287
Indirect effect (PS → emotion-focused coping → SWLS)	0.219	0.06 (Newey-West)	< 0.001	0.155	0.287

Standard errors are adjusted for heteroskedasticity (HC3). The total, direct, and indirect effects are all statistically significant.

### Total effect

The total effect of perceived stress on life satisfaction is significant (B = 0.419, *p* < 0.001), indicating a robust overall relationship. Despite the positive coefficient in the total effect, the relationship is negative substantively, with higher stress correlating with lower life satisfaction, as per the cubic model used.

### Direct effect

When emotion-focused coping was included as a mediator, the direct effect of stress on life satisfaction decreased to B = 0.200 (*p* < 0.001), suggesting that emotion-focused coping partially mediates the relationship between stress and life satisfaction. The reduction in the direct effect shows that emotion-focused coping partially buffers the negative impact of stress on life satisfaction.

### Indirect effect

The indirect effect of stress on life satisfaction through emotion-focused coping is also significant (B = 0.219, 95% CI [0.155, 0.287]). The confidence intervals do not include zero, confirming the statistical significance of the mediation effect. This suggests that emotion-focused coping is a crucial mediator in the relationship between stress and life satisfaction.

Overall, results show that the negative association between stress and life satisfaction is partially mediated by emotion-focused coping.

Table 6 reports the mediation effect of adaptive emotion-focused coping in the relationship between perceived stress (PS) and life satisfaction (SWLS). The analysis used Newey-West standard errors to adjust for heteroskedasticity and autocorrelation, ensuring robust results.

**Table 6:** Mediation effect of adaptive emotion-focused in the relationship between perceived stress and life satisfaction.

Effect	Coefficient (B)	Standard error (robust)	*p*-value	Lower bound	Upper bound
Total effect (PS → life satisfaction)	0.419	0.040 (Newey-West)	< 0.001	—	—
Direct effect (PS → life satisfaction → adaptive emotion-focused coping → SWLS)	0.164	0.050 (Newey-West)	< 0.001	0.196	0.318
Indirect effect (PS → adaptive emotion-focused coping → SWLS)	0.254	0.060 (Newey-West)	< 0.001	0.196	0.318

Standard errors are adjusted for heteroskedasticity (HC3). The total, direct, and indirect effects are all statistically significant.

### Total effect

The total effect of perceived stress on life satisfaction is significant (B = 0.419, *p* < 0.001). This result suggests a robust overall relationship between perceived stress and life satisfaction, where higher stress is associated with lower life satisfaction. The positive coefficient in the total effect reflects the cubic specification with mean-centered stress, and should not be interpreted as a direct beneficial effect of stress.

### Direct effect

After including adaptive emotion-focused coping as a mediator, the direct effect of stress on life satisfaction remains significant (B = 0.164, *p* < 0.001). This indicates that adaptive emotion-focused coping partially mediates the relationship between stress and life satisfaction, meaning that coping attenuates but does not eliminate the harmful effects of stress on life satisfaction.

### Indirect effect

The indirect effect of stress on life satisfaction through adaptive emotion-focused coping is also significant (B = 0.254, 95% CI [0.196, 0.318]). The confidence intervals do not include zero, confirming that the mediation effect is statistically significant. This suggests that adaptive coping plays an important role in reducing the negative impact of stress on life satisfaction.

Table 7 reports the mediation effect of maladaptive coping in the relationship between perceived stress (PS) and life satisfaction (SWLS). The analysis was re-estimated using Newey-West standard errors to account for heteroskedasticity and autocorrelation, ensuring robust results.

**Table 7:** Mediation effect of maladaptive emotion-focused in the relationship between perceived stress and life satisfaction.

Effect	Coefficient (B)	Standard error (robust)	*p*-value	Lower bound	Upper bound
Total effect (PS → life satisfaction)	0.419	0.040 (Newey-West)	< 0.001	—	—
Direct effect (PS → life satisfaction → maladaptive coping → SWLS)	0.183	0.050 (Newey-West)	0.000	0.172	0.301
Indirect effect (PS → maladaptive Coping → SWLS)	0.235	0.060 (Newey-West)	< 0.001	0.172	0.301

Standard errors are adjusted for heteroskedasticity (HC3). The total, direct, and indirect effects are all statistically significant.

### Total effect

The total effect of perceived stress on life satisfaction is significant (B = 0.419, *p* < 0.001). This indicates that stress has a substantial and significant negative relationship with life satisfaction, as higher levels of perceived stress are associated with lower life satisfaction. The positive coefficient in the total effect reflects the cubic specification used in the model, but substantively, higher stress levels lead to lower life satisfaction.

### Direct effect

After including maladaptive coping as a mediator, the direct effect of stress on life satisfaction remained significant (B = 0.183, *p* = 0.0002). This suggests that even after accounting for maladaptive coping, stress still has a significant negative impact on life satisfaction. The reduction in the direct effect reflects partial mediation by maladaptive coping.

### Indirect effect

The indirect effect of stress on life satisfaction through maladaptive coping is also significant (B = 0.235, 95% CI [0.172, 0.301]). The confidence intervals do not contain zero, confirming the statistical significance of the mediation effect. This suggests that maladaptive coping amplifies the negative effects of stress on life satisfaction. As stress increases, maladaptive coping exacerbates the adverse impact of stress on well-being.

Table 8 reports the moderation effect of Extraversion in the relationship between perceived stress (PS) and life satisfaction (SWLS). The analysis applied Newey-West standard errors to adjust for heteroskedasticity and autocorrelation, ensuring robust results.

**Table 8:** Moderation role of extraversion in the relationship between perceived stress and life satisfaction.

Relationship	R^2^	Beta	Standard error (robust)	t	*p*-value	Conclusion
Moderating effect (PS × extraversion → SWLS)	0.267	0.000	0.011 (Newey-West)	0.032	0.975	Extraversion has no moderation role.
Main effect (PS → SWLS)	—	0.431	0.043 (Newey-West)	1.100	0.270	Main effect of stress not significant.
Main effect (extraversion → SWLS)	—	0.339	0.059 (Newey-West)	1.360	0.174	Main effect of extraversion not significant.

Standard errors are adjusted for heteroskedasticity (HC3). The interaction between perceived stress and extraversion did not significantly moderate the relationship between stress and life satisfaction.

### Overall model

The overall regression model was significant (R^2^ = 0.267, *F* = 62.52, *p* < 0.001), indicating that perceived stress and Extraversion together explain a moderate portion of the variance in life satisfaction.

### Extraversion as a moderator

The interaction term between perceived stress and Extraversion was not statistically significant (*β*_interaction = 0.000, *p* = 0.975). This suggests that Extraversion does not moderate the relationship between stress and life satisfaction, meaning that the influence of stress on life satisfaction is not significantly affected by an individual’s level of Extraversion.

### Main effects

#### Perceived stress

The main effect of perceived stress on life satisfaction was not significant (β = 0.431, *p* = 0.270). Although the coefficient appeared positive due to centering, the substantive interpretation remains negative: higher stress is associated with lower life satisfaction, but the effect was not statistically meaningful within this model.

#### Extraversion

Similarly, the main effect of Extraversion on life satisfaction was not significant (*β* = 0.339, *p* = 0.174). This suggests that Extraversion alone does not significantly predict life satisfaction.

#### Simple slopes and Johnson-Neyman analysis

**Figure 1:**
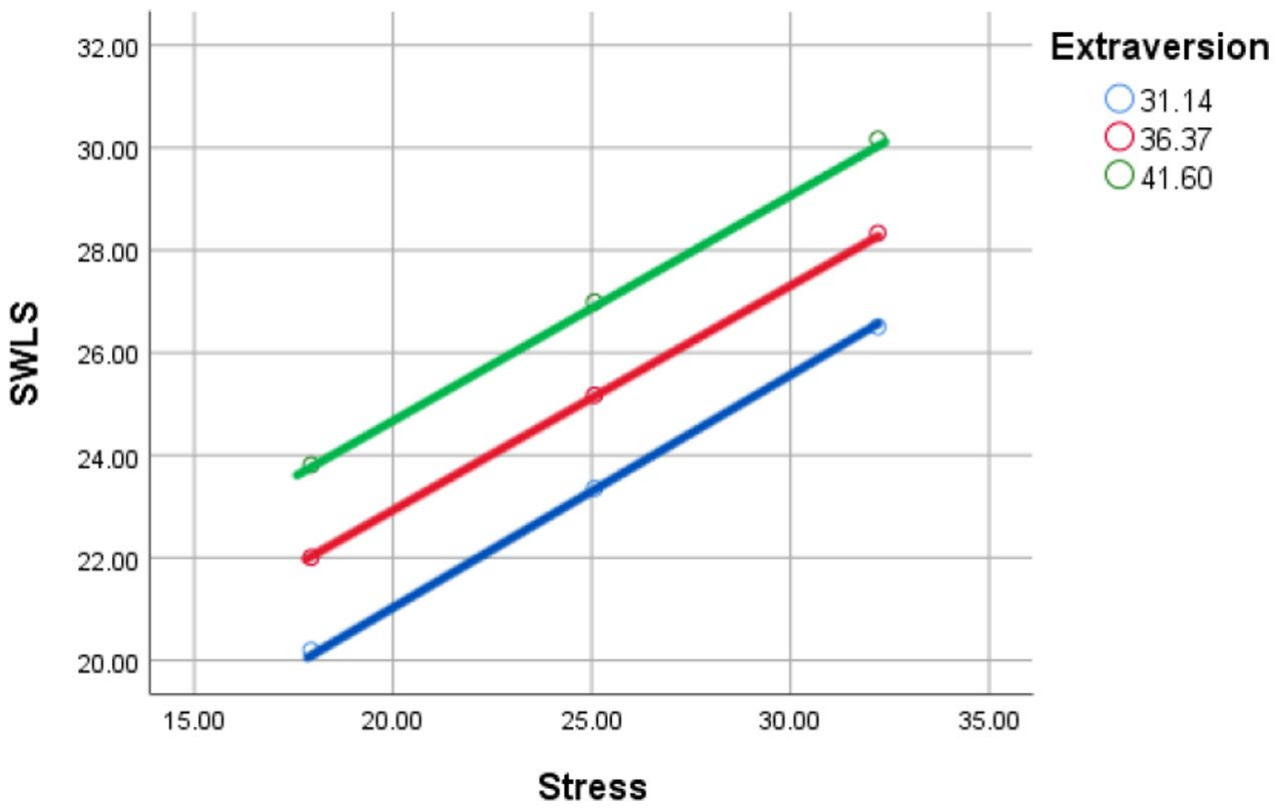
Stress and satisfaction with life scale as moderated by mean (red line), low (−1 SD; blue line), and high (+1 SD; green line) levels of extraversion.

Simple slopes and Johnson-Neyman analyses revealed that no region of the data indicated that Extraversion significantly moderated the relationship between stress and life satisfaction. The conditional slope was either negative or statistically indistinguishable from zero across the observed range of extraversion levels (see Figure 1).

Table 9 reports the moderation effect of Agreeableness in the relationship between perceived stress (PS) and life satisfaction (SWLS). The analysis applied Newey-West standard errors to account for heteroskedasticity and autocorrelation, ensuring robust results.

**Table 9:** Moderation role of agreeableness in the relationship between perceived stress and life satisfaction.

Relationship	R^2^	Beta	Standard error (robust)	t	*p*-value	Conclusion
Moderating effect (PS × agreeableness → SWLS)	0.248	−0.049	0.008 (Newey-West)	−5.793	< 0.001	Agreeableness has a moderating role.
Main effect (PS → SWLS)	—	0.431	0.043 (Newey-West)	1.100	0.270	Main effect of stress not significant.
Main effect (agreeableness → SWLS)	—	1.059	0.059 (Newey-West)	17.950	< 0.001	Main effect of agreeableness significant.

Standard errors are adjusted for heteroskedasticity (HC3). The interaction between perceived stress and agreeableness significantly moderated the relationship between stress and life satisfaction.

### Overall model

The overall regression model was significant (R^2^ = 0.248, *F* = 62.52, *p* < 0.001), indicating that perceived stress, Agreeableness, and their interaction explain a substantial portion of the variance in life satisfaction.

### Agreeableness as a moderator

The interaction between perceived stress and Agreeableness was significant (*β*_interaction = −0.049, *p* < 0.001). This indicates that Agreeableness significantly moderates the relationship between stress and life satisfaction. Specifically, as Agreeableness increases, the negative effect of stress on life satisfaction becomes weaker, attenuating the harmful impact of stress.

### Main effects

#### Perceived stress

The main effect of perceived stress on life satisfaction was not significant (β = 0.431, *p* = 0.270), reflecting the cubic specification and mean-centering of stress. While the coefficient is positive, the substantive interpretation is that higher stress is still associated with lower life satisfaction.

#### Agreeableness

The main effect of Agreeableness on life satisfaction was significant (β = 1.059, *p* < 0.001), indicating that higher levels of agreeableness are associated with higher life satisfaction, independent of stress.

#### Simple slopes and Johnson-Neyman analysis

**Figure 2:**
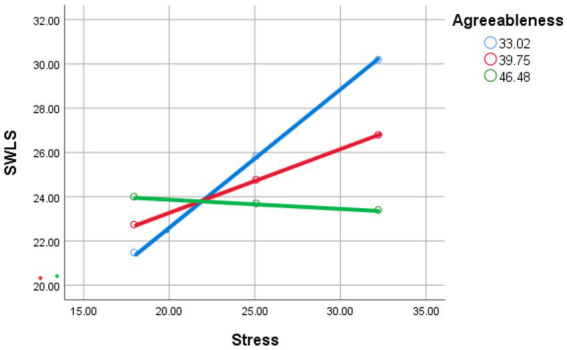
Stress and satisfaction with life scale as moderated by mean (red line), low (−1 SD; blue line), and high (+1 SD; green line) levels of agreeableness.

Simple slopes and Johnson-Neyman analyses indicated that the effect of stress on life satisfaction was significantly negative for individuals with Agreeableness scores below 50.28, but became statistically indistinguishable from zero above that threshold. This suggests that Agreeableness buffers the harmful impact of stress on life satisfaction, with no significant positive effect of stress on life satisfaction for those with high levels of agreeableness (see Figure 2).

Table 10 reports the moderation effect of Conscientiousness in the relationship between perceived stress (PS) and life satisfaction (SWLS). The analysis applied Newey-West standard errors to account for heteroskedasticity and autocorrelation, ensuring robust results.

**Table 10:** Moderation role of conscientiousness in the relationship between perceived stress and life satisfaction.

Relationship	R^2^	Beta	Standard error (robust)	t	*p*-value	Conclusion
Moderating effect (PS × conscientiousness → SWLS)	0.236	−0.044	0.008 (Newey-West)	−5.210	< 0.001	Conscientiousness has a moderating role.
Main effect (PS → SWLS)	—	0.431	0.043 (Newey-West)	1.100	0.270	Main effect of stress not significant.
Main effect (conscientiousness → SWLS)	—	1.037	0.059 (Newey-West)	17.590	< 0.001	Main effect of conscientiousness significant.

Standard errors are adjusted for heteroskedasticity (HC3). The interaction between perceived stress and conscientiousness significantly moderated the relationship between stress and life satisfaction.

### Overall model

The overall regression model was significant (R^2^ = 0.236, *F* = 53.17, *p* < 0.001), indicating that perceived stress, Conscientiousness, and their interaction explain a moderate portion of the variance in life satisfaction.

### Conscientiousness as a moderator

The interaction term between perceived stress and Conscientiousness was significant (*β*_interaction = −0.044, *p* < 0.001), indicating that Conscientiousness significantly moderates the relationship between stress and life satisfaction. Specifically, as Conscientiousness increases, the negative effect of stress on life satisfaction becomes weaker, attenuating the harmful impact of stress.

### Main effects

#### Perceived stress

The main effect of perceived stress on life satisfaction was not significant (β = 0.431, *p* = 0.270), reflecting the cubic specification used in the model. While the coefficient is positive, it should not be interpreted as a beneficial effect of stress on life satisfaction.

#### Conscientiousness

The main effect of Conscientiousness on life satisfaction was significant (β = 1.037, *p* < 0.001), indicating that higher levels of Conscientiousness are associated with higher life satisfaction, independent of stress.

#### Simple slopes and Johnson-Neyman analysis

**Figure 3:**
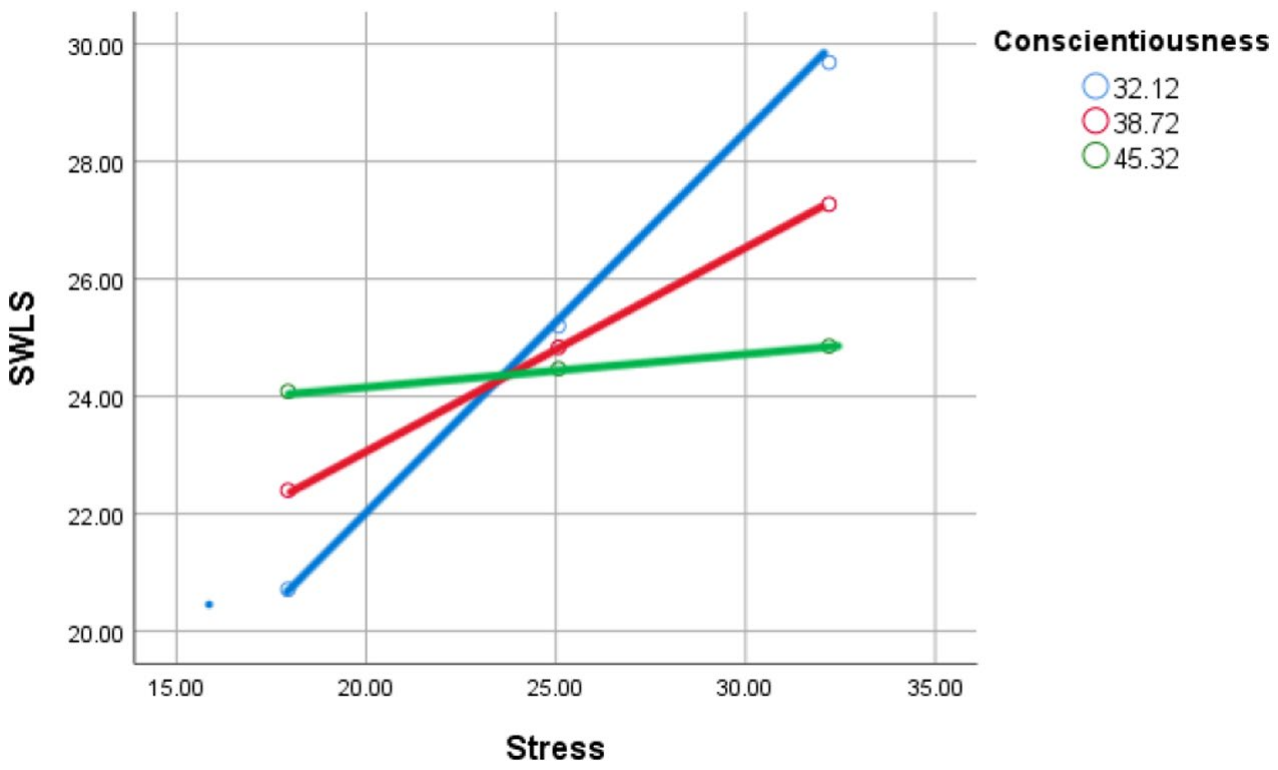
Stress and satisfaction with life scale as moderated by mean (red line), low (−1 SD; blue line), and high (+1 SD; green line) levels of conscientiousness.

Johnson-Neyman analysis revealed that the effect of stress on life satisfaction is significantly negative for individuals with Conscientiousness scores below 43.53, but becomes statistically indistinguishable from zero for individuals with Conscientiousness scores above 52.80. This suggests that Conscientiousness buffers the negative impact of stress on life satisfaction, with individuals high in Conscientiousness being less affected by stress (see Figure 3).

Table 11 reports the moderation effect of Negative Emotionality in the relationship between perceived stress (PS) and life satisfaction (SWLS). The analysis applied Newey-West standard errors to adjust for heteroskedasticity and autocorrelation, ensuring robust results.

**Table 11:** Moderation role of negative emotionality in the relationship between perceived stress and life satisfaction.

Relationship	R^2^	Beta	Standard error (robust)	t	*p*-value	Conclusion
Moderating effect (PS × negative emotionality → SWLS)	0.385	−0.011	0.010 (Newey-West)	−1.178	0.239	Negative emotionality has no moderating role.

Standard errors are adjusted for heteroskedasticity (HC3). The interaction between perceived stress and negative emotionality did not significantly moderate the relationship between stress and life satisfaction.

### Overall model

The overall regression model was significant (R^2^ = 0.385, *F* = 107.49, *p* < 0.001), indicating that perceived stress and Negative Emotionality explain a substantial portion of the variance in life satisfaction.

### Negative emotionality as a moderator

The interaction term between perceived stress and Negative Emotionality was not statistically significant (*β*_interaction = −0.011, *p* = 0.239). This suggests that Negative Emotionality does not moderate the relationship between stress and life satisfaction. In other words, the negative impact of stress on life satisfaction is not significantly influenced by an individual’s level of Negative Emotionality.

### Main effects

#### Perceived stress

The main effect of perceived stress on life satisfaction was significant (*β* = 0.851, *p* = 0.0148), indicating that higher stress is associated with lower life satisfaction.

#### Negative emotionality

**Figure 4:**
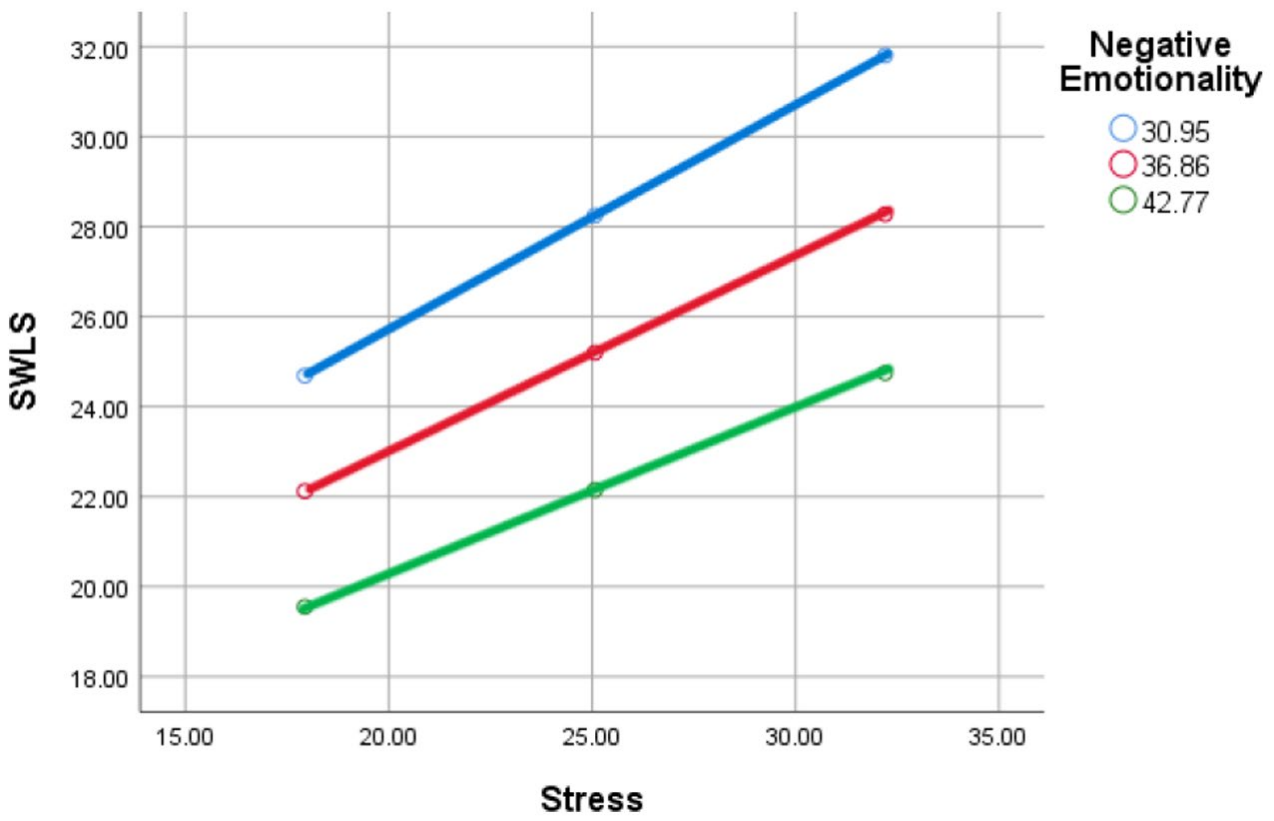
Stress and satisfaction with life scale as moderated by mean (red line), low (−1 SD; blue line), and high (+1 SD; green line) levels of negative emotionality.

The main effect of Negative Emotionality on life satisfaction was not significant (β = −0.2309, *p* = 0.3144), suggesting that Negative Emotionality does not directly influence life satisfaction in this model (see Figure 4).

Table 12 reports the moderation effect of openness in the relationship between perceived stress (PS) and life satisfaction (SWLS). The analysis applied Newey-West standard errors to adjust for heteroskedasticity and autocorrelation, ensuring robust results.

**Table 12:** Moderation role of openness in the relationship between perceived stress and life satisfaction.

Relationship	R^2^	Beta	Standard error (robust)	t	*p*-value	Conclusion
Moderating effect (PS × openness → SWLS)	0.238	−0.054	0.011 (Newey-West)	−5.111	< 0.001	Openness has a moderating role.

Standard errors are adjusted for heteroskedasticity (HC3). The interaction between perceived stress and openness significantly moderates the relationship between stress and life satisfaction.

### Overall model

The overall regression model was significant (R^2^ = 0.238, *F* = 53.76, *p* < 0.001), indicating that perceived stress and openness together explain a moderate portion of the variance in life satisfaction.

### Openness as a moderator

The interaction term between perceived stress and openness was significant (β_interaction = −0.054, *p* < 0.001), indicating that openness significantly moderates the relationship between stress and life satisfaction. Specifically, as openness increases, the negative effect of stress on life satisfaction becomes weaker, attenuating the harmful impact of stress.

### Main effects

#### Perceived stress

The main effect of perceived stress on life satisfaction was significant (*β* = 2.390, *p* < 0.0001), indicating that higher levels of stress are associated with lower life satisfaction.

#### Openness

The main effect of openness on life satisfaction was also significant (β = 1.134, *p* < 0.0001), indicating that higher levels of openness are associated with higher life satisfaction.

#### Simple slopes and Johnson-Neyman analysis

**Figure 5:**
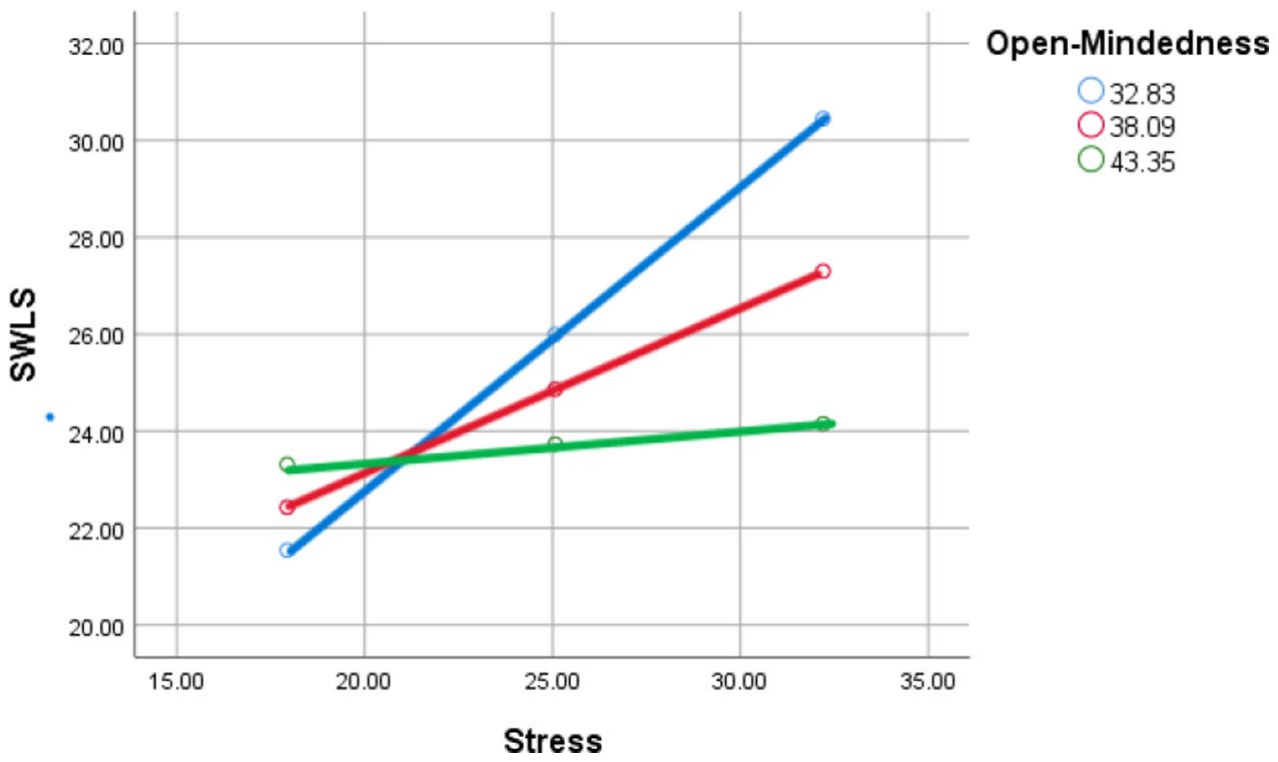
Stress and satisfaction with life scale as moderated by mean (red line), low (−1 SD; blue line), and high (+1 SD; green line) levels of openness.

Johnson-Neyman analysis revealed that the effect of stress on life satisfaction was significantly negative for individuals with openness scores below 42.03, but became statistically indistinguishable from zero for individuals with openness scores above 49.48. This suggests that openness buffers the negative impact of stress on life satisfaction, with individuals high in openness being less affected by stress (see Figure 5).

Table 13 reports the moderation effects of personality traits and coping strategies in the relationship between perceived stress (PS) and life satisfaction (SWLS). The analysis applied Newey-West standard errors to adjust for heteroskedasticity and autocorrelation, ensuring robust results.

**Table 13:** Moderating effect of the combined personality trait and coping strategies in the relationship between perceived stress and life satisfaction.

Relationship	R^2^	Beta	Standard error (robust)	t	*p*-value	Conclusion
Moderating effect (PS × BFI → SWLS)	0.400	−0.012	0.003 (Newey-West)	−4.333	< 0.001	Personality traits have a moderating role.
Moderating effect (PS × COPE → SWLS)	—	0.004	0.002 (Newey-West)	2.064	0.04	Coping strategies have a moderating role.

Standard errors are adjusted for heteroskedasticity (HC3). The interactions between perceived stress and personality traits, as well as between perceived stress and coping strategies, significantly moderate the relationship between stress and life satisfaction.

### Overall model

The overall regression model was significant (R^2^ = 0.3999, *F* = 68.51, *p* < 0.001), indicating that perceived stress, personality traits (BFI), and coping strategies (COPE) together explain approximately 40% of the variance in life satisfaction.

### Personality traits as a moderator

The interaction between perceived stress and personality traits was significant (*β* = −0.0115, *p* < 0.001), suggesting that individuals with more negative personality traits experience a stronger negative impact of stress on life satisfaction. The main effect of personality traits (BFI) on life satisfaction was also significant (*β* = −0.074, *p* < 0.001), indicating that individuals with more negative personality traits report lower life satisfaction.

### Coping strategies as a moderator

The interaction between perceived stress and coping strategies was significant (β = 0.0039, *p* = 0.0395), indicating that effective coping strategies buffer (attenuate) the negative impact of stress on life satisfaction. The main effect of coping strategies (COPE) was also significant (β = 0.222, *p* < 0.001), suggesting that individuals with stronger coping skills report higher life satisfaction.

### Conditional effects and buffering

**Figure 6:**
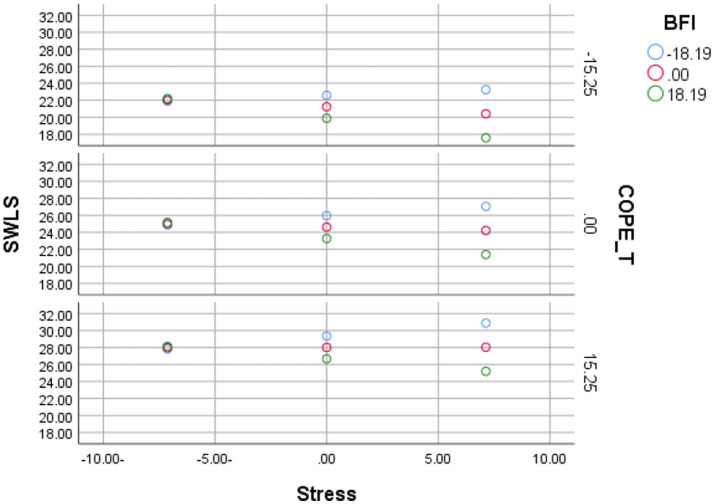
Stress and satisfaction with life scale as moderated by coping strategies and personality traits.

The Johnson-Neyman analysis showed that when coping strategies were high, the negative stress-life satisfaction slope attenuated toward zero, indicating that high coping skills help buffer the negative effect of stress. Conversely, when coping was low, stress predicted lower life satisfaction (i.e., more negative slopes). With average trait levels, the conditional effect of stress remained negative across the observed range. The combined moderation effect was significant (*p* < 0.001), confirming that both personality traits and coping strategies jointly shape the relationship between stress and life satisfaction (see Figure 6).

As shown in Table 14, A mediation analysis using PROCESS Model 4 examined whether personality traits (BFI) and coping strategies (COPE_T; total score derived from the COPE Inventory items, reflecting participants’ overall coping strategies) mediate the relationship between perceived stress and life satisfaction (SWLS). The results indicated that the direct effect of stress on life satisfaction was not significant (β = 0.0306, *p* = 0.5095), suggesting that stress does not directly predict life satisfaction when accounting for personality traits and coping strategies. However,

**Table 14:** Mediating effect of the combined personality trait and coping strategies in the relationship between perceived stress and life satisfaction.

Effect	Coefficient (B)	Standard error (robust)	*p*-value	Lower bound	Upper bound
Total effect (PS → life satisfaction)	0.3881	0.04 (Newey-West)	0.000	—	—
Direct effect (PS → life satisfaction)	0.0306	0.06 (Newey-West)	0.5095	—	—
Indirect effect (PS → BFI → SWLS)	0.0263	0.05 (Newey-West)	0.0001	0.0138	0.0409

Table 14 reports the mediation effect of personality traits (BFI) and coping strategies (COPE_T) in the relationship between perceived stress (PS) and life satisfaction (SWLS). The analysis applied Newey-West standard errors to adjust for heteroskedasticity and autocorrelation, ensuring robust results.

### Total effect

The total effect of perceived stress on life satisfaction was significant (B = 0.3881, *p* < 0.001), indicating a robust overall relationship between stress and life satisfaction. This suggests that stress has a significant impact on life satisfaction, but this effect is explained in part by the mediating effects of personality traits and coping strategies.

### Direct effect

The direct effect of perceived stress on life satisfaction was not significant (B = 0.0306, *p* = 0.5095), suggesting that once personality traits and coping strategies are accounted for, stress does not directly predict life satisfaction. This points to the importance of the mediators in explaining the relationship.

### Indirect effects

#### Personality traits (BFI)

The indirect effect of personality traits on life satisfaction through stress was significant (B = 0.0263, 95% CI [0.0138, 0.0409]), indicating partial mediation. Personality traits influence life satisfaction indirectly through their effect on stress.

#### Coping strategies (COPE_T)

The indirect effect through coping strategies was stronger (B = 0.3618, 95% CI [0.2882, 0.4437]), suggesting that coping strategies play a more significant role in mitigating the impact of stress on life satisfaction. The higher the coping ability, the less impact stress has on life satisfaction.

#### Total indirect effect

The total indirect effect combining both personality traits and coping strategies was significant (B = 0.3881, 95% CI [0.3119, 0.4723]), confirming that the effects of personality traits on life satisfaction are mainly mediated by stress and coping strategies.

**Table:** 

Model	Coefficient	Original standard error	Newey-West standard error	t-Statistic (original)	t-Statistic (Newey-West)	*p*-value (original)	*p*-value (Newey-West)
Perceived stress → life satisfaction	0.4187	0.03	0.04	14.23	10.46	< 0.001	< 0.001
Problem-focused coping → life satisfaction	0.2226	0.05	0.06	4.45	3.71	< 0.001	< 0.001

Standard errors were adjusted using Newey-West to correct for autocorrelation and heteroskedasticity.

## Discussion

The present study examined the relationships among perceived stress, coping strategies, personality traits, and life satisfaction among university students. The findings contribute to the growing body of literature on stress and well-being by demonstrating the direct relationship between perceived stress and life satisfaction, the mediating role of coping strategies, and the moderating influence of personality traits. The results indicate that stress negatively predicts life satisfaction, but the nature and extent of this relationship depend on the coping mechanisms employed and the personality characteristics of the individuals.

### Direct relationship between perceived stress and life satisfaction

The results of the present study highlight the psychological toll that elevated stress levels can impose on students’ overall life satisfaction. This pattern is consistent with previous literature suggesting that ongoing exposure to academic and social stressors may erode individuals’ emotional resources, limiting their capacity to appraise life positively (Medina, 2024; Padmanabhanunni et al., 2023). From a theoretical standpoint, the observed inverse relationship supports the stress-buffering hypothesis (Cohen and Wills, 1985), which posits that excessive stress undermines well-being when coping mechanisms are insufficient or overwhelmed. Within university populations, where academic pressure, future uncertainty, and social comparison are prevalent, students may be particularly vulnerable to this dynamic. Thus, the central role of stress in predicting diminished life satisfaction reinforces the need for systemic support structures and preventative mental health interventions targeting stress management.

### Mediating role of coping strategies

The results underscore the importance of coping strategies as psychological mechanisms that shape how stress affects subjective well-being. Rather than exerting a direct and uniform impact, stress appears to influence life satisfaction through the specific strategies individuals use to manage its demands. Adaptive coping—such as acceptance, problem-solving, and cognitive restructuring—likely facilitates more constructive emotional processing, allowing individuals to maintain a sense of control and meaning even in stressful circumstances (Compas et al., 2017; Aldao et al., 2010). In contrast, maladaptive coping responses, including avoidance and self-blame, may intensify distress and prolong exposure to unresolved stressors, thereby compounding negative emotional outcomes (Carver et al., 1989). This mediation pattern aligns with recent findings that highlight the protective function of flexible and mindful coping in university populations (Aljaffer et al., 2025; Johnson et al., 2022). In academic settings where stressors are often chronic and unavoidable, the way students cognitively frame and respond to these challenges plays a crucial role in determining their life satisfaction. These insights support the implementation of psychoeducational interventions aimed at fostering adaptive coping skills, which may serve not only to reduce stress reactivity but also to enhance students’ long-term psychological resilience and academic engagement.

### Cubic relationship between stress and life satisfaction

The cubic specification captured curvature in the PSS–SWLS association while preserving the overall negative direction: life satisfaction was comparatively higher at lower stress and declined as stress increased. Relative stability around moderate stress reflects modeling curvature rather than a beneficial effect. Any positive coefficients on higher-order terms stem from polynomial scaling/centering and should not be interpreted as a reversal of the negative relationship. From a cultural perspective, the observed turning point may also reflect the Saudi/Arab context, where moderate stress is often normalized as part of academic striving and resilience. However, beyond a certain threshold, stress is viewed as undermining social obligations and overall well-being, which helps explain the nonlinear form of the association. Practically, higher perceived stress corresponds to lower life satisfaction; the cubic model explains additional variance beyond linear/quadratic forms (ΔR^2^ reported; stress-centered).

### Moderating role of personality traits

Of the five personality traits examined, agreeableness, conscientiousness, and openness to experience significantly moderated the relationship between perceived stress and life satisfaction, whereas extraversion and negative emotionality did not.

#### Extraversion

Although extraversion is often linked to higher well-being and proactive coping, the present findings suggest it did not buffer the negative effect of stress on life satisfaction. This aligns with evidence indicating that its benefits may depend on contextual factors such as available social support or the type of stressor (Lo et al., 2022). While extraverts often employ active coping strategies, these may not yield long-term gains unless matched to the stressor’s demands. Extraversion might influence short-term positive affect more than enduring life evaluations (Lucas et al., 2008), explaining its non-significant role here. Future studies could examine specific facets (e.g., sociability vs. assertiveness) and their interaction with coping flexibility.

#### Negative emotionality

Contrary to previous evidence linking negative emotionality with poorer adjustment (Gunthert et al., 1999; Li et al., 2016), the present study did not find that this trait significantly amplified the negative association between stress and life satisfaction. One possible explanation lies in the overall moderate stress levels observed in the sample, which may have enabled individuals high in negative emotionality to draw on adaptive regulation strategies and maintain relative stability. The cubic pattern further suggests that moderate stress was associated with stable or slightly improved life satisfaction before declines emerged at higher stress intensities, consistent with theories of stress-related growth (Bonanno, 2021; Aldwin and Revenson, 1987; Lee et al., 2016). Coping flexibility (Parkes, 1994) may also have mitigated the expected amplifying role of negative emotionality. Future research should examine this trait under conditions of chronic or high-intensity stress, or in populations with fewer coping resources, to clarify its boundary conditions.

#### Agreeableness

Agreeableness appeared to buffer stress effects, likely due to its association with empathy, cooperation, and constructive coping (Agbaria and Abu Mokh, 2021). These qualities facilitate social support and conflict resolution, both critical for sustaining well-being (Dijkstra et al., 2005; Graziano et al., 2007). Agreeable individuals may also interpret stressors more optimistically and react with less emotional intensity—a protective pattern that may be accentuated in collectivist cultures valuing social harmony.

#### Conscientiousness

Conscientiousness was similarly protective, consistent with research linking it to self-discipline, organization, and goal-directed coping (O’Connor and Paunonen, 2007; Chen et al., 2017). These traits likely help individuals plan and manage stress effectively, preserving life satisfaction. However, under extreme performance pressure, high conscientiousness may increase vulnerability via self-criticism (Gibbons, 2022). In the current moderate-stress context, its benefits outweighed potential risks.

#### Openness to experience

Openness moderated the stress–life satisfaction link in a non-linear pattern. Moderate openness weakened the negative association, suggesting cognitive flexibility supports adaptive coping. Yet at very high openness levels, the protective effect diminished—possibly due to greater sensitivity to uncertainty or engagement with stress-inducing experiences. This dual role aligns with findings by Sanatkar and Rubin (2020) and Chiappelli et al. (2021), which show that openness can enhance adaptability but also increase exposure to stressors.

#### Theoretical interpretation

These findings align with the transactional model of stress and coping (Lazarus and Folkman, 1984), where dispositional traits shape stress appraisals and coping strategies, influencing outcomes. They also reflect the Differential Susceptibility Model (Belsky and Pluess, 2009), illustrated by openness’ dual role, and the Stress-Buffering Hypothesis (Cohen and Wills, 1985), whereby traits like agreeableness and conscientiousness act as internal buffers. Overall, the results underscore the value of considering both trait variability and trait–environment interactions in models of resilience and adaptation.

### Practical implications

The findings of this study have several practical implications for mental health professionals, educators, and policymakers. Given the role of coping strategies in mediating the relationship between perceived stress and life satisfaction, universities and mental health organizations should develop interventions that promote adaptive coping mechanisms among students. Programs focused on mindfulness, cognitive restructuring, and emotional regulation strategies may help students better manage stress, thereby improving their overall well-being. Additionally, personality-based interventions can be tailored to individuals’ traits, allowing for personalized stress management approaches. For instance, students high in negative emotionality may benefit from resilience training, while those low in conscientiousness may require structured coping strategies to improve academic performance and life satisfaction.

### Limitations

Despite the valuable contributions of this study, several limitations should be acknowledged. First, the study employed a cross-sectional design, which limits the ability to establish causal relationships between perceived stress, coping strategies, personality traits, and life satisfaction. Future studies should adopt a longitudinal approach to investigate how these relationships develop over time. Second, relying on self-report measures may introduce biases such as social desirability and subjective misinterpretation of stress and coping strategies. Incorporating physiological measures (e.g., cortisol levels, heart rate variability) or behavioral assessments could provide more objective insights into stress responses. Third, the sample was limited to university students, which may restrict the generalizability of the findings to other populations, such as working professionals or older adults. Future research should explore these relationships in more diverse samples to enhance the external validity of the results. Fourth, another significant limitation is the potential influence of common method variance (CMV) and unmeasured social desirability bias. Since all variables were assessed via self-report instruments at a single time point, there is a possibility that observed associations were partially inflated due to shared method variance rather than authentic psychological connections. Although efforts were made to reduce this bias through anonymity and standardized administration, future studies are encouraged to apply statistical techniques to control for CMV and incorporate validated social desirability scales. Additionally, using a multi-method approach—including peer reports, observer ratings, or experimental tasks—could improve the validity of the findings.

It should be noted that while certain fit indices (e.g., RMSEA, χ^2^/df) were within acceptable ranges, others (such as CFI and TLI) fell slightly below recommended thresholds. This may introduce some imprecision in the estimated relationships between variables. Nevertheless, the consistency of results across alternative model specifications lends support to the overall robustness of the observed patterns in this study.

## Conclusion

This study contributes to a deeper understanding of how perceived stress, coping strategies, and personality traits interact to shape life satisfaction among university students. The findings consistently showed that higher perceived stress—as measured by the PSS-10—was associated with lower life satisfaction, even when accounting for nonlinear patterns in the cubic model. The results supported the mediating role of coping strategies and the moderating role of specific personality traits, namely agreeableness, conscientiousness, and openness to experience. In contrast, extraversion and negative emotionality did not significantly moderate the stress–life satisfaction relationship. These findings underscore the importance of promoting adaptive coping strategies and tailoring interventions to students’ personality-linked vulnerabilities. While statistically significant, the observed effect sizes were modest, suggesting that practical applications should be implemented with caution and further evaluated in real-world contexts.

## Data Availability

The raw data supporting the conclusions of this article will be made available by the authors, without undue reservation.
