# Supplementary material for: Perceived stress and life satisfaction among university students: the mediating and moderating roles of coping strategies and personality traits
Source: Front Psychol. 2025 Sep 23;16:1593555. doi: 10.3389/fpsyg.2025.1593555 (PMC12500562; doi:10.3389/fpsyg.2025.1593555)
Supplement: Supplementary file 1 [file Supplementary_file_1.docx]

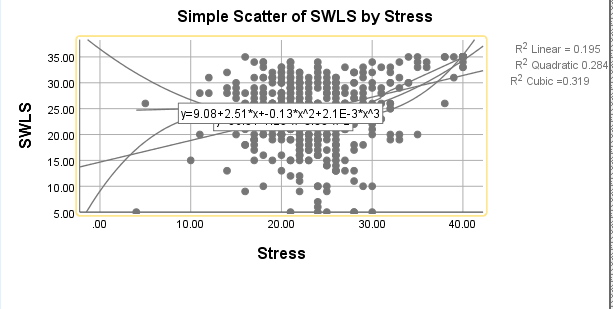


**Figure A.** Comparison of linear, quadratic, and cubic models of the relationship between perceived stress (centered PSS-10 scores) and life satisfaction (SWLS). While the linear model explained 19.5% of the variance (R² = .195), the quadratic model improved fit (R² = .284, ΔR² = .089).
