# Supplementary material for: Perceived stress and life satisfaction among university students: the mediating and moderating roles of coping strategies and personality traits
Source: Front Psychol. 2025 Sep 23;16:1593555. doi: 10.3389/fpsyg.2025.1593555 (PMC12500562; doi:10.3389/fpsyg.2025.1593555)
Supplement: Supplementary file 2 [file Table_1.docx]

| **Scale** | **Chi (DoF)** | **CFI** | **TLI** | **SRMR** | **RMSEA (90% CI)** |
| --- | --- | --- | --- | --- | --- |
| **PSS** | 102.47 (35) | 0.953 | 0.932 | 0.0476 | 0.051 (0.042 to 0.058) |
| **Brief COPE** | 1517.96 (347) | 0.776 | 0.756 | 0.083 | 0.081 (0.076 to 0.085) |
| **SWLS** | 21.08 (5) | 0.982 | 0.963 | 0.026 | 0.079 (0.046 to 0.115) |
| **BFI** | 6527.34 (1642) | 0.621 | 0.605 | 0.096 | 0.076 (0.074 to 0.078) |

Supplementary Table A: Model fit indices for construct validity of the scales used.
